# Pharmaco‐genetic inhibition of pyramidal neurons retards hippocampal kindling‐induced epileptogenesis

**DOI:** 10.1111/cns.13434

**Published:** 2020-06-28

**Authors:** Li‐Ying Chen, Jiao Liang, Fan Fei, Ye‐Ping Ruan, He‐Ming Cheng, Yi Wang, Zhong Chen, Ceng‐Lin Xu

**Affiliations:** ^1^ Institute of Pharmacology & Toxicology NHC and CAMS Key Laboratory of Medical Neurobiology College of Pharmaceutical Sciences Zhejiang University Hangzhou China; ^2^ Department of Neurology Epilepsy Center Second Affiliated Hospital School of Medicine Zhejiang University Hangzhou China; ^3^ College of Pharmaceutical Science Zhejiang Chinese Medical University Hangzhou China

**Keywords:** epilepsy, parvalbumin neurons, pharmaco‐genetics, pyramidal neurons, synaptic plasticity

## Abstract

**Aims:**

Pharmaco‐genetics emerges as a new promising approach for epileptic seizures. Whether it can modulate epileptogenesis is still unknown.

**Methods:**

Here, parvalbumin neurons and pyramidal neurons of the seizure focus were transfected with engineered excitatory Gq‐coupled human muscarinic receptor hM3Dq and engineered inhibitory Gi‐coupled human muscarinic receptor hM4Di, respectively. And their therapeutic value in mouse hippocampal kindling‐induced epileptogenesis was tested.

**Results:**

Pharmaco‐genetic activating parvalbumin neurons limitedly retarded the progression of behavioral seizure stage and afterdischarge duration (ADD) during epileptogenesis induced by kindling. Activating parvalbumin neurons delayed seizure development only in the early stage, but accelerated it in late stages. On the contrary, pharmaco‐genetic inhibiting pyramidal neurons robustly retarded the progression of seizure stages and ADDs, which greatly delayed seizure development in both early and late stages. Although both pharmaco‐genetic therapeutics efficiently alleviated the severity of acute kindling‐induced seizures, pharmaco‐genetic inhibiting pyramidal neurons were able to reverse the enhanced synaptic plasticity during epileptogenesis, compared with that of pharmaco‐genetic activating parvalbumin neurons.

**Conclusion:**

Our results demonstrated that pharmaco‐genetic inhibiting pyramidal neurons retard hippocampal kindling‐induced epileptogenesis and reverse the enhanced synaptic plasticity during epileptogenesis, compared with that of pharmaco‐genetic activating parvalbumin neurons. It suggests that pharmaco‐genetics targeting pyramidal neurons may be a promising treatment for epileptogenesis.

## Graphical abstract

Pharmaco‐genetic activating parvalbumin neurons of the seizure focus limitedly retarded the progression of behavioral seizure stage and afterdischarge duration (ADD) during epileptogenesis in hippocampal kindling mice. On the contrary, Pharmaco‐genetic inhibiting pyramidal neurons of the seizure focus robustly retarded epileptogenesis.

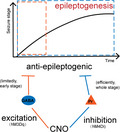

## 1 INTRODUCTION

Epilepsy, which is pathologically characterized by sudden unexpected hypersynchronous discharges with abnormal neuronal excitability affects more than 60 million people globally.^1^ Temporal lobe epilepsy (TLE) is one of the most common forms of epilepsy, in which epileptic seizures usually initiate locally within the hippocampus and may secondarily spread out to the entire brain.^2^ It is often medically refractory from a clinical therapeutic standpoint due to its frequent resistance to antiepileptic drugs and surgical resection.^3^, ^4^, ^5^ In addition, although neuromodulation using vagus nerve stimulation, transcranial magnetic stimulation, or deep brain stimulation has become new options for epilepsy treatment,^6^ the responder rate is still relatively low^7^ and inconsistent results are often produced,^8^ possibly due to the non‐specific effect. Poor control of epileptic seizures together with unexpected injuries caused by seizures and other complications brings heavy burdens to the patients.^9^

Recently, selective modulation of neuronal excitability by Designer Receptors Exclusively Activated by Designer Drugs (DREADD)‐based pharmaco‐genetic technology represents a valuable approach for disease therapeutics,^10^, ^11^ including epilepsy.^12^ Epilepsy is considered as a circuit‐level syndrome characterized by “excitation‐inhibition” imbalance that largely depends on the interaction of excitatory glutamatergic pyramidal neurons and inhibitory GABAergic neurons.^13^ Many previous studies demonstrated that pharmaco‐genetic inhibition of pyramidal neurons by engineered inhibitory Gi‐coupled human muscarinic receptor hM4Di alleviated seizure severity in different types of epilepsy models in vitro and in vivo.^14^, ^15^, ^16^, ^17^ Meanwhile, we previously demonstrated that pharmaco‐genetic activating parvalbumin neurons (a subtype of GABAergic neuron), with engineered excitatory Gq‐coupled human muscarinic receptor hM3Dq, also produced seizure attenuation,^18^ which may be a novel and promising approach for treating refractory TLE. Later study showed that pharmaco‐genetic activating parvalbumin interneurons exhibited the better effect in reducing epileptiform activity than the other subtypes of GABAergic neurons.^19^ These studies suggest that seizure attenuation by pharmaco‐genetic through targeting either parvalbumin neurons or pyramidal neurons is likely to be an efficient approach for treating epilepsy. However, current pharmaco‐genetics for epilepsy treatment all focus on seizure control. Whether it can be used to modulate epileptogenesis, a highly dynamic process from normal state to a reduced threshold for seizure or the propensity to generate spontaneous seizures,^20^ is still uninvestigated.

Therefore, in this study, we aim to investigate the therapeutic value of pharmaco‐genetic activating parvalbumin neurons in hippocampal kindling‐induced epileptogenesis, compared with pharmaco‐genetic inhibiting pyramidal neurons.

## 2 MATERIALS AND METHODS

### 2.1 Animals

*CaMKII2α‐Cre* (stock number: 005359) and *PV‐Cre* (stock number: 008069) mice were used and genotyped according to the protocols of Jackson Laboratory. Wildtype mice were negative littermates. Three to four months old male mice were used in this study. The mice were raised in cages with a 12‐hour light/dark cycle in groups prior to surgery (lights on from 8:00 to 20:00). Foods and water were provided at libitum. Behavioral experiments were performed between 9:00 and 18:00. The use and care of the mice were in accordance of the ethical guidelines of the Zhejiang University Animal Experimentation Committee and the National Institutes of Health Guide for the Care and Use of Laboratory Animals.

### 2.2 Viral delivery

Viral delivery procedures were performed strictly according to our previous study.^18^ Briefly, for pharmaco‐genetic activating the parvalbumin neurons, 0.5‐μL Cre‐inducible adeno‐associated virus (AAV‐EF1α‐DIO‐hM3Dq‐mCherry) was stereotactically microinjected into the right ventral hippocampus (AP, −2.9 mm; ML, −3.0 mm; and V, −3.0 mm) of the *PV‐Cre* mice based on the mouse brain atlas (named *PV‐hM3Dq* mice). For pharmaco‐genetic inhibiting the pyramidal neurons, 0.5‐μL AAV‐EF1α‐DIO‐hM4Di‐mCherry was stereotactically microinjected into the right ventral hippocampus of the *CaMKII2α‐Cre* mice (named *CaMKII2α‐hM4Di* mice). Viral suspension was injected by an injection pump (Micro4, World Precision Instruments) with a 1‐μL syringe at 100 nL/min. The needle was left in place for 5 minutes after each injection to prevent backflow and then slowly withdrawn. Mice were kept for at least 4 weeks before further experiments to allow the expression of virus. All the viruses were purchased from the Neuron Biotech Co and stored at −80°C until use.

### 2.3 Hippocampal kindling‐induced epileptogenesis

Under sodium pentobarbital anesthesia (50 mg/kg, i.p.), the mice were mounted in the stereotaxic apparatus (512600, Stoelting), and then prefabricated bipolar electrodes for kindling stimulation and EEG recording were implanted into the ventral hippocampal (AP: −2.9 mm; ML: −3.1 mm; V: −3.1 mm). The bipolar electrodes were made of twisted stainless steel wires (diameter 0.125 mm, AM Systems) with a 0.5 mm tip separation. The reference and ground were connected to two screws which were placed in the skull over the cerebellum. The coordinates were measured from Bregma according to the atlas of mouse brain.^21^ After all the behavioral studies, the location of electrodes was histologically verified in all animals.

Mice were allowed to recover for 1 week after surgery, then kindling acquisition was performed as our previous studies.^22^, ^23^ Initially, the afterdischarge threshold (ADT), which is the indicator of baseline epileptogenic susceptibility, of each mouse was determined. The stimulation intensity was started at 40 μA and was then increased by an additional 20 μA every 1 minute. The minimal intensity that produced at least a 5‐second ADD was defined as the ADT for that animal and was used for grouping thereafter. Subsequently, every mouse received 10 kindling stimulations (400 μA, 20 Hz, 2‐second trains, 1‐ms monophasic square‐wave pulses) daily with 30 minutes intervals, induced by a constant‐current stimulator (SEN‐7203, SS‐202J; Nihon Kohden) and ADD was recorded with a Neuroscan system (Compumedics). Behavioral seizure severity was classified into different stages according to the Racine classification^24^ and scored by a double‐blind experienced experimenter. Mice usually showed the seizure stage 1‐5: 1, mouth and facial movement; 2, head nodding; 3, forelimb clonus; 4. rearing with forelimb clonus; 5, rearing and falling with forelimb clonus. Stages 1‐3 were considered to be focal seizures and stages 4‐5 as generalized seizures. Mice were regarded as fully kindled until they showed three consecutive stage 5 seizures.

To investigate the effect of pharmaco‐genetic modulation on the kindling acquisition, the Clozapine N‐oxide (CNO) was injected once daily (i.p., 1.0 mg/kg) 0.5 hour before the first electrical stimulation. We recorded the seizure EEG, seizure stage, and ADD after each kindling stimulation. The power spectral densities of the seizure EEG induced by 30th kindling stimulation were derived by using the fast Fourier transform algorithm of the Neuroscan EEG analysis software package as our previous study,^25^ and the absolute power within different frequency bands was calculated: *δ* (delta, 0‐4 Hz); *θ* (theta, 4‐8 Hz); *α* (alpha, 8‐12 Hz); *β* (beta, 12‐30 Hz); *γ* (gamma, 30‐100 Hz); and total power (0‐100 Hz).^23^, ^26^, ^27^

### 2.4 In vivo single‐unit recordings and analysis

To verify whether the virus was functional in vivo, neuronal spikes were recorded and analyzed in *PV‐hM3Dq* and *CaMKII2α‐hM4Di* mice which were anesthetized by urethane as our previous studies.^28^ Briefly, 8‐wire bundle of microelectrodes (12 μm, AM‐Systems) with an impedance of 1‐2 MΩ was used for recording. The neuronal activity was sampled by the Cerebus acquisition system with a sample rate of 30 kHz and high‐pass filtered at 250 Hz (Blackrock Microsystems), which was referenced online against a wire within the same brain area that had a signal‐to‐noise ratio >3:1. A screw above the cerebellum was used as ground. When the electrode reached the target brain region, baseline single‐unit activity was recorded, and then CNO was given (i.c.v., 2 μL, 5 μmol/L) to test its interference with the neural firing rate.

To test the neuronal response in hippocampal seizure, single‐unit recordings were performed and analyzed in urethane‐anesthetized wildtype mice. Putative pyramidal neurons and GABAergic neurons were distinguished by three independent criteria (firing rate, spike width, and autocorrelation pattern) based on previous studies.^29^, ^30^ Spikes of putative pyramidal neurons were identified by the low firing rate (≤10 Hz), wide spike waveform (≥0.3 ms), and sharp autocorrelation, whereas spikes of GABAergic neurons were identified by the high firing rate (>5 Hz), narrow spike waveform (≤0.30 ms), and flat autocorrelation. An “excited” or “inhibited” neuronal response was defined as follows: firing rates were considered to be significantly different if they were >2 standard deviations (SDs), greater or less than baseline averages.

### 2.5 Field excitatory postsynaptic potential (fEPSP) tests

For fEPSP test,^31^ electrodes were implanted into the stratum pyramidale of ventral CA3 (AP: −2.9 mm; ML: −3.1 mm; V: −3.1 mm) for electric stimulation and the stratum radiatum of CA1 field (AP, −3.4 mm; ML, −2.0 mm; V, −1.8 mm) for fEPSP recording, respectively. Test stimulations (single 0.1‐ms monophasic square‐wave pulses) were delivered through Nihon Kohden stimulator every 10‐second (0.1 Hz) with varying intensities (100‐800 μA) to the ventral hippocampal CA3, the corresponding evoked field potentials were recorded in the CA1 with PowerLab system (AD Instruments). Then, input/output curve the maximum population spike amplitude was determined for each individual mouse and all potentials employed as baseline criteria were evoked at a stimulus intensity. The test pulse was determined as the stimulation intensity that produced a 50% of maximum response of the population spike (usually 200‐600 μA). The population spike amplitude was measured by averaging the amplitude from the peak to the base of fEPSPs. The results of the fEPSP tests for before and after kindling acquisition and with or without pharmaco‐genetic modulation were compared.

### 2.6 Immunohistochemistry

After behavioral tests, mice were then deeply anesthetized with pentobarbital (100 mg/kg, intraperitoneal) and transcardially perfused with saline followed by 4% paraformaldehyde in a 0.1 mol/L phosphate buffer (Sigma‐Aldrich). The brains were removed and post‐fixed in the 4% phosphate‐buffered paraformaldehyde at 4°C overnight, and were then cryo‐protected with 30% (w/v) sucrose. Coronal 30‐μm slices of brains were cut on a freezing sliding microtome (Leica). Brain slices were processed for immunofluorescence for PV (1:1000, Swant PV27), then incubated with primary antibody diluted in phosphate‐buffered saline with 0.15% Triton X‐100 overnight at 4°C. Then they were rinsed with phosphate‐buffered saline and incubated with an Alexa‐488 conjugated secondary fluorescent antibody (1:400, Jackson Immuno Research) at 1 μg/mL for 2 hours at room temperature. The slices were mounted on slides by the Vectashield Mounting Media (Vector Labs) after rinsing, and the immunofluorescence was accessed by using an Olympus microscope (BX61).

### 2.7 Statistics

Data are presented as the mean ± SEM. Numbers of experimental replicates (n) were indicated in figure legend. Statistical analysis was performed by Prism 8.0 with appropriate inferential methods as indicated in the figure legends. Statistical tests were applied after testing for normal distribution (Shapiro‐Wilk test). Two‐way ANOVA followed by post hoc Tukey test analysis was used for multiple comparisons and unpaired Student's *t* test was used for two group comparisons. No statistical methods were used to pre‐determine sample size, or to randomize. Only a two‐tailed *P* value < .05 was considered statistically significant.

## 3 RESULTS

### 3.1 Pharmaco‐genetic activating parvalbumin neurons limitedly retard hippocampal kindling‐induced epileptogenesis

**FIGURE 1:**
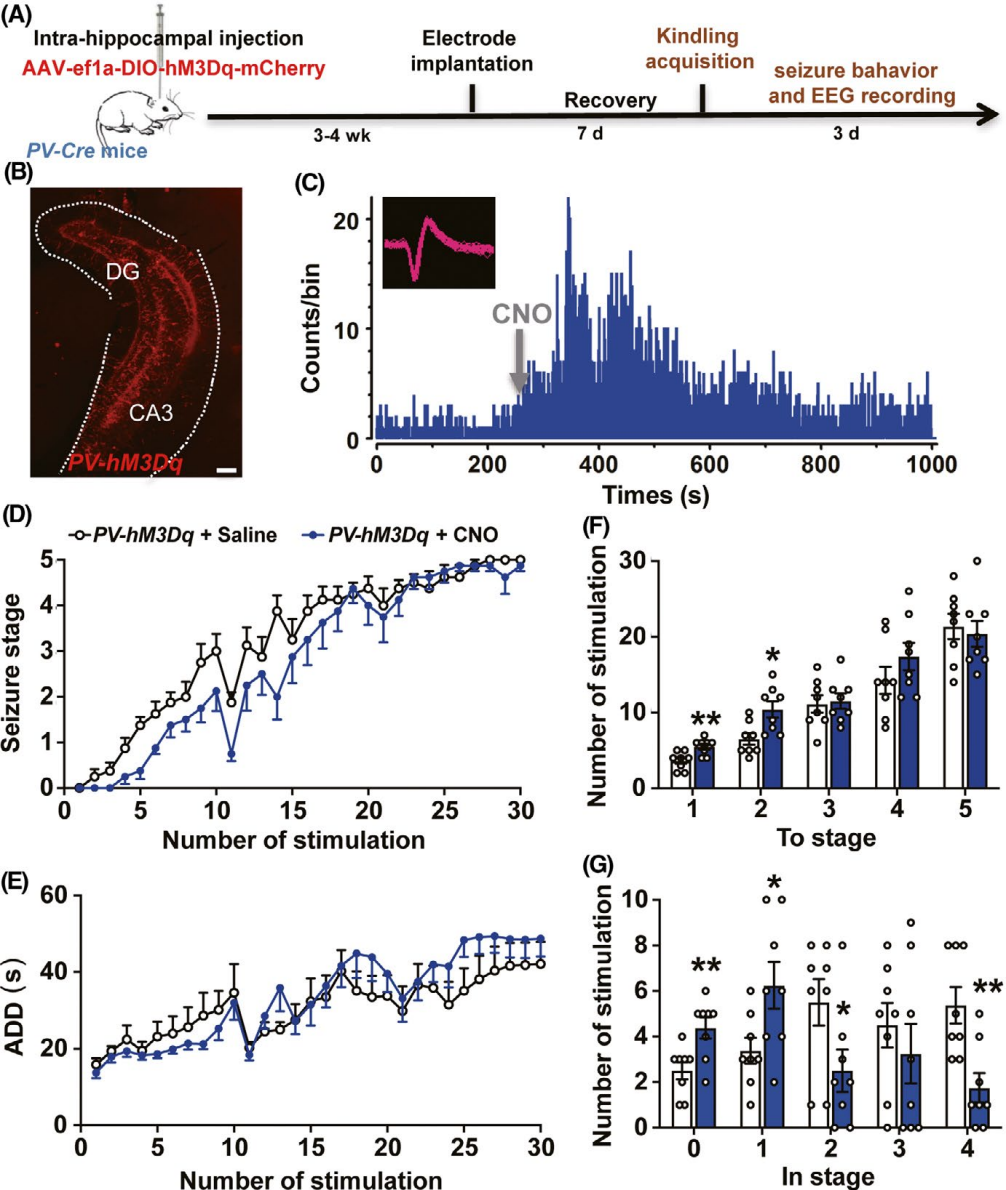
Pharmaco‐genetic activating parvalbumin neurons limitedly retards hippocampal kindling‐induced epileptogenesis. A, Experiment scheme for pharmaco‐genetic activating parvalbumin neurons in hippocampal kindling‐induced epileptogenesis model. B, Representative image of the hippocampal hM3Dq‐mCherry expression from a *PV‐hM3Dq* mouse; scale bar 100 μm. C, Representative peri‐event raster histogram for the firing of a putative parvalbumin neuron before and after the CNO treatment in a *PV‐hM3Dq* mouse (10‐ms bins). Insert, spike waveform of a putative parvalbumin neuron. D, E, The effects of pharmaco‐genetic activating parvalbumin neurons on the progression of seizure stage (D) and afterdischarge duration (ADD) (E) in hippocampal kindling‐induced epileptogenesis model. n = 8 for each group, two‐way ANOVA test was used. F, G, Number of kindling stimulations required to reach each seizure stage (F) and stay at each seizure stage (G) during hippocampal kindling‐induced epileptogenesis. n = 8 for each group, *P < .05, **P < .01, compared with Saline group, unpaired *t* test was used. Data are presented as means ± SEM

To investigate the effects of pharmaco‐genetic activating parvalbumin neurons in hippocampal kindling‐induced epileptogenesis, a Cre‐inducible adeno‐associated virus, AAV‐EF1α‐DIO‐hM3Dq‐mCherry were stereotactically microinjected into the right ventral hippocampus of PV‐Cre mice (named PV‐hM3Dq mice, Figure 1A). Immunohistochemical results demonstrated that hM3Dq‐mCherry was widely expressed in hippocampal CA3 and dentate gyrus (DG) (Figure 1B). To test whether the hM3Dq‐expressed parvalbumin neurons were functional, single‐unit recordings were performed in the ventral hippocampal CA3. As parvalbumin neuron, one subtype of GABAergic neuron, is characterized by fast‐spiking electrophysiological feature, we sorted putative GABAergic neurons based on narrow half‐width spike, high firing rates, and the sharp autocorrelogram.^29^, ^30^ We found that CNO injection reliably increased the firing rate in 4 of 6 putative GABAergic neurons (Figure 1C), indicating that CNO treatment can activate the parvalbumin neurons in the ventral hippocampus.

In kindling model, CNO was injected 0.5 hour before the first kindling stimulation each day in *PV‐hM3Dq* mice, and we found that it nearly had no obvious effect on the progression of behavioral seizure stages and ADD, compared with the saline group (two‐way ANOVA test, *F* = 2.777, *P* = .1177 for seizure stage; *F* = 0.3491, *P* = .5640 for ADD; Figure 1D,E). To analyze the stepwise progression of kindling acquisition, the number of stimulations required to reach and stay at each seizure stage was calculated. We found that the number of stimulations required to reach the focal seizure stages 1 and 2 was increased by CNO treatment (unpaired *t* test, *t* = 3.319, *df* = 14, *P* = .0051 for To stage 1; *t* = 2.962, *df* = 14, *P* = .0103 for To stage 2; Figure 1F), and this could be caused by the increased number of stimulations to stay in seizure stage 0 and 1 (unpaired *t* test, *t* = 3.147, *df* = 14, *P* = .0071 for In stage 0; *t* = 2.446, *df* = 14, *P* = .0283 for In stage 1; Figure 1G). Interestingly, the number of stimulations to stay in seizure stage 2 and 4 was even decreased by CNO treatment (unpaired *t* test, *t* = 2.181, *df* = 14, *P* = .0468 for in stage 2; *t* = 3.520, *df* = 14, *P* = .0034 for in stage 4; Figure 1G). Thus, above data suggested that pharmaco‐genetic activating parvalbumin neurons limitedly retard hippocampal kindling‐induced epileptogenesis, if any in early stages.

### 3.2 Pharmaco‐genetic inhibiting pyramidal neurons efficiently retard hippocampal kindling‐induced epileptogenesis

**FIGURE 2:**
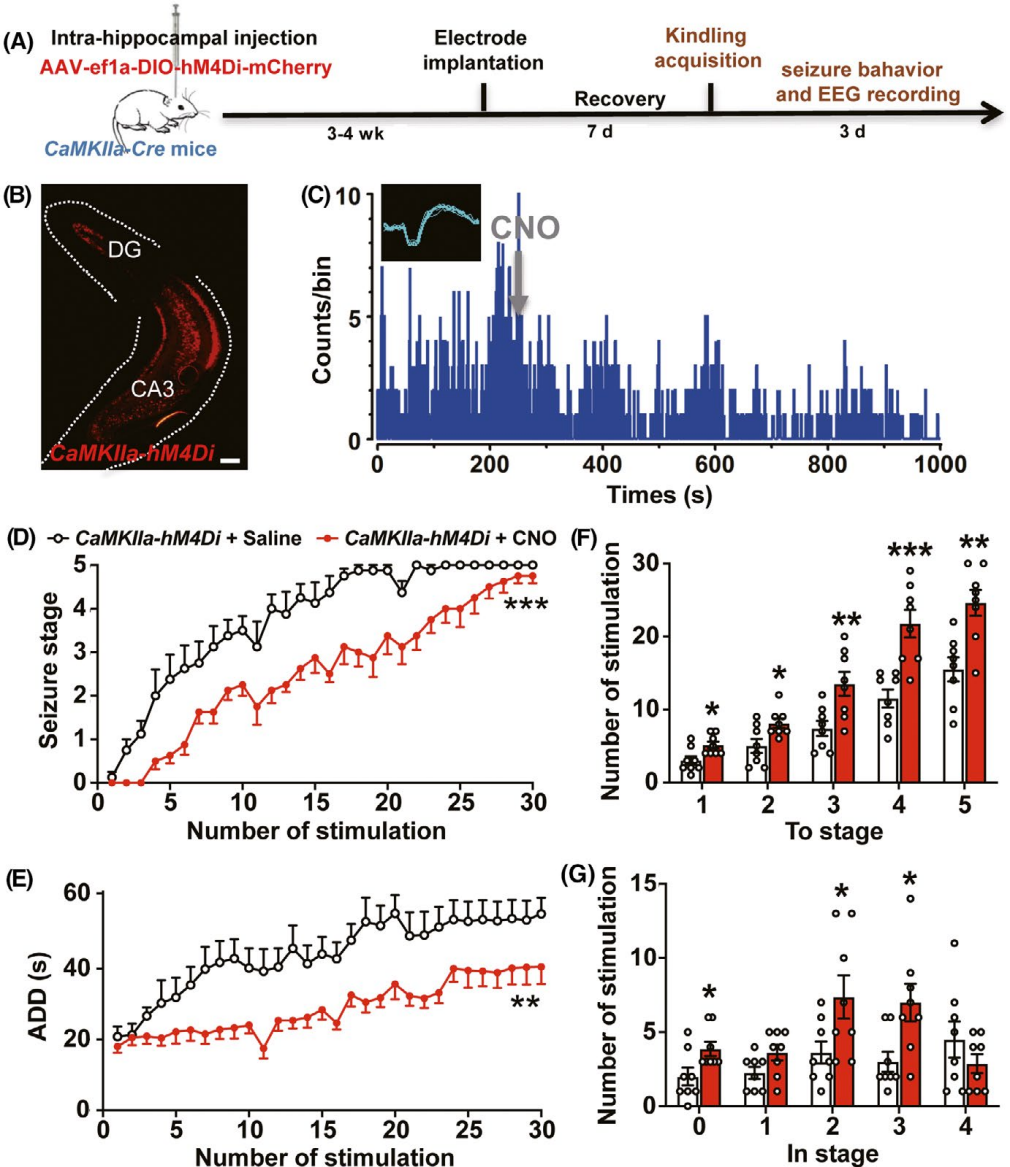
Pharmaco‐genetic inhibiting pyramidal neurons efficiently retards hippocampal kindling‐induced epileptogenesis. A, Experiment scheme for pharmaco‐genetic inhibiting pyramidal neurons in hippocampal kindling‐induced epileptogenesis model. B, Representative image of the hippocampal hM4Di‐mCherry expression from a *CaMKIIa‐hM4Di* mouse; scale bar 100 μm. C, Representative peri‐event raster histogram for the firing of a putative pyramidal neuron before and after the CNO treatment in a *CaMKIIa‐hM4Di* mouse (10‐ms bins). Insert, spike waveform of a putative pyramidal neuron. D, E, The effects of pharmaco‐genetic inhibiting pyramidal neurons on the progression of seizure stage (D) and afterdischarge duration (ADD) (E) in hippocampal kindling‐induced epileptogenesis model. n = 8 for each group, **P < .01, ***P < .001, two‐way ANOVA test was used. F, G, Number of kindling stimulations required to reach each seizure stage (F) and stay at each seizure stage (G) during hippocampal kindling‐induced epileptogenesis. n = 8 for each group, *P < .05, **P < .01, ***P < .001, compared with Saline group, unpaired *t* test was used. Data are presented as means ± SEM

Next, we investigated whether pharmaco‐genetic inhibiting pyramidal neurons would retard hippocampal kindling‐induced epileptogenesis. The Cre‐inducible adeno‐associated virus AAV‐EF1α‐DIO‐hM4Di‐mCherry was injected into the right ventral hippocampus of *CaMKIIa*‐Cre mice (name *CaMKIIa‐hM4Di* mice, Figure 2A). Immunohistochemical results demonstrated that hM4Di‐mCherry was mainly expressed in the CA3 and DG (Figure 2B). Single‐unit recordings showed that CNO treatment decreased the firing rate of putative pyramidal neurons (8/11) in *CaMKIIa‐hM4Di* mice (Figure 2C), indicating that pyramidal neurons in the ventral hippocampus can be inhibited by CNO. In kindling model, the progression of behavioral seizure stages and ADD were significantly retarded by CNO treatment, compared with saline in *CaMKIIa‐hM4Di* mice (two‐way ANOVA test, *F* = 22.76, *P* = .0003 for seizure stage; *F* = 10.16, *P* = .0016 for ADD; Figure 2D,E). CNO treatment also increased the number of stimulations required to reach all the seizure stages (unpaired *t* test, For To stage 1, *t* = 2.773, *df* = 14, *P* = .0149; For To stage 2, *t* = 2.667, *df* = 14, *P* = .0184; For To stage 3, *t* = 3.164, *df* = 14, *P* = .0069; For To stage 4, *t* = 4.524, *df* = 14, *P* = .0005; For To stage 5, *t* = 3.748, *df* = 14, *P* = .0022; Figure 2F), and increased number of stimulations to stay in stage 1, 2 and 3 (unpaired *t* test, For In stage 0, *t* = 2.447, *df* = 14, *P* = .0282; For In stage 2, *t* = 2.278, *df* = 14, *P* = .0389; For In stage 3, *t* = 2.779, *df* = 14, *P* = .0148; Figure 2G). Thus, above data indicated that pharmaco‐genetic inhibiting pyramidal neurons efficiently retards hippocampal kindling‐induced epileptogenesis.

**FIGURE 3:**
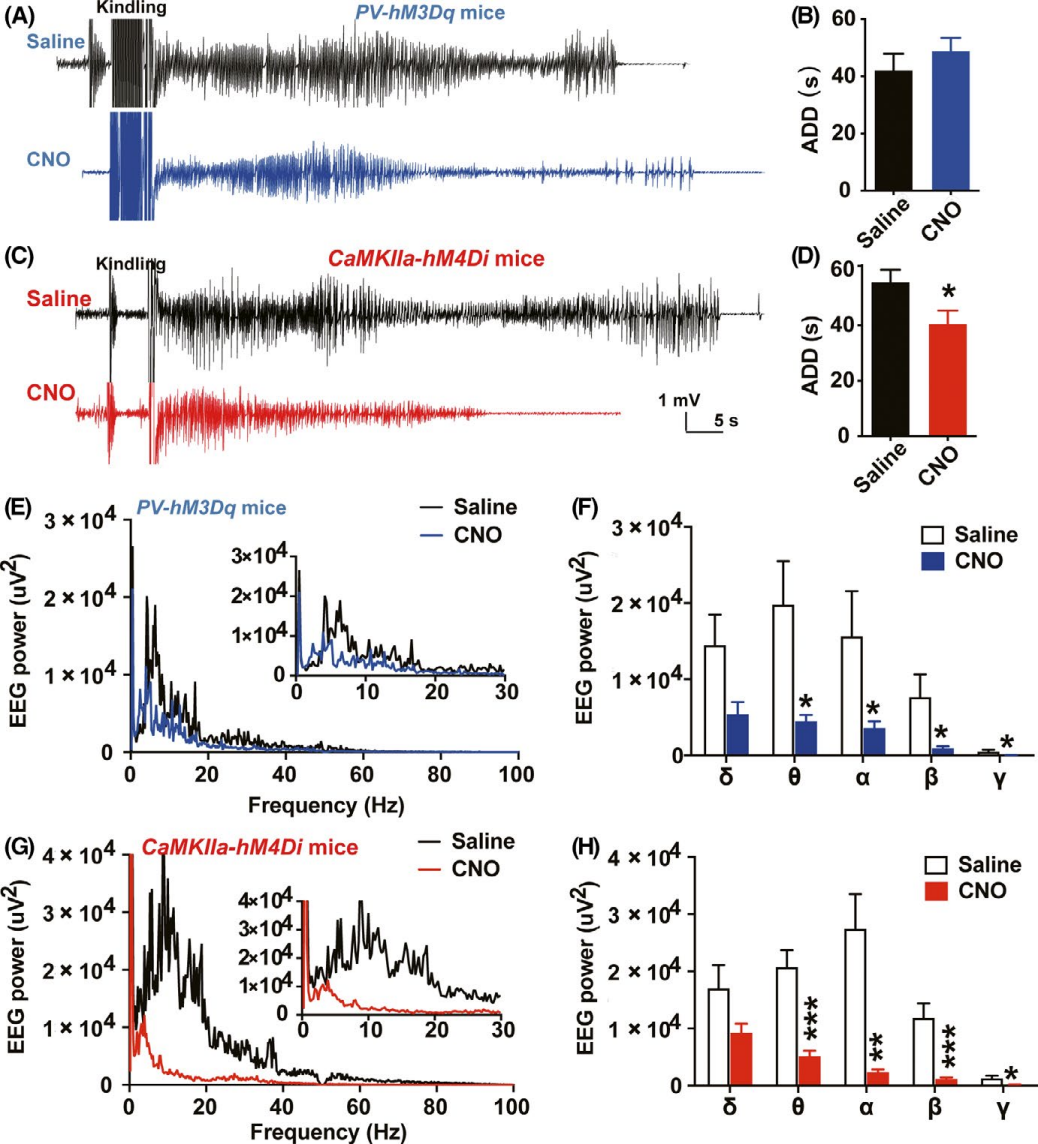
Pharmaco‐genetic inhibiting pyramidal neurons efficiently alleviate the severity of kindling‐induced seizures. A, B, Representative EEGs (A) and statistics of afterdischarge duration (ADD) (B) recorded from the hippocampus at the 30th stimulation during epileptogenesis from *PV‐hM3Dq* mice. n = 8 for each group, unpaired *t* test was used. C, D, Representative EEGs (C) and statistics of ADD (D) recorded from the hippocampus at the 30th stimulation during epileptogenesis from *CaMKIIa‐hM4Di* mice. n = 8 for each group, *P < .05, compared with Saline group, unpaired *t* test was used. E, F, The effects of pharmaco‐genetic activating parvalbumin neurons on the EEG spectrum analysis (E) and statistics of each EEG rhythm (F) during hippocampal seizures in *PV‐hM3Dq* mice. n = 8 for each group, *P < .05, compared with Saline group, unpaired *t* test was used. G, H, The effects of pharmaco‐genetic activating parvalbumin neurons on the EEG spectrum analysis (G) and statistics of each EEG rhythm (H) during hippocampal seizures in *CaMKIIa‐hM4Di* mice. n = 8 for each group, *P < .05, **P < .01, ***P < .001, compared with Saline group, unpaired *t* test was used. Data are presented as means ± SEM

Furthermore, we tested the effects of pharmaco‐genetic activating parvalbumin neurons on EEG intensity of hippocampal seizures and compared with the effect caused by pharmaco‐genetic inhibiting pyramidal neurons. We found that pharmaco‐genetic activating parvalbumin neurons showed no effect on the ADD of hippocampal seizures (unpaired *t* test, *t* = 0.8936, *df* = 14, *P* = .3867; Figure 3A,B), but slightly alleviated the EEG intensity (local field potential) of hippocampal seizures (unpaired *t* test, For *δ*, *t* = 2.136, *df* = 14, *P* = .0508; For *θ*, *t* = 2.670, *df* = 14, *P* = .0183; For *α*, *t* = 2.028, *df* = 14, *P* = .0310; For *β*, *t* = 2.264, *df* = 14, *P* = .0400; For *γ*, *t* = 2.798, *df* = 14, *P* = .0142; Figure 3E,F). While, pharmaco‐genetic inhibition of pyramidal neurons not only significantly shortened the duration of hippocampal seizures (unpaired *t* test, *t* = 2.261, *df* = 14, *P* = .0402; Figure 3C,D), but also efficiently alleviated the EEG intensity of hippocampal seizures (unpaired *t* test, For *δ*, *t* = 1.824, *df* = 14, *P* = .0895; For *θ*, *t* = 5.073, *df* = 14, *P* = .0002; For *α*, *t* = 4.156, *df* = 14, *P* = .0010; For *β*, *t* = 4.270, *df* = 14, *P* = .0008; For *γ*, *t* = 2.838, *df* = 14, *P* = .0131; Figure 3G,H). Thus, all above results indicated that pharmaco‐genetic inhibiting pyramidal neurons much more efficiently retards hippocampal kindling‐induced epileptogenesis than that of pharmaco‐genetic activating parvalbumin neurons.

### 3.3 Pharmaco‐genetic inhibiting pyramidal neurons efficiently depotentiates hippocampal synaptic plasticity during epileptogenesis

**FIGURE 4:**
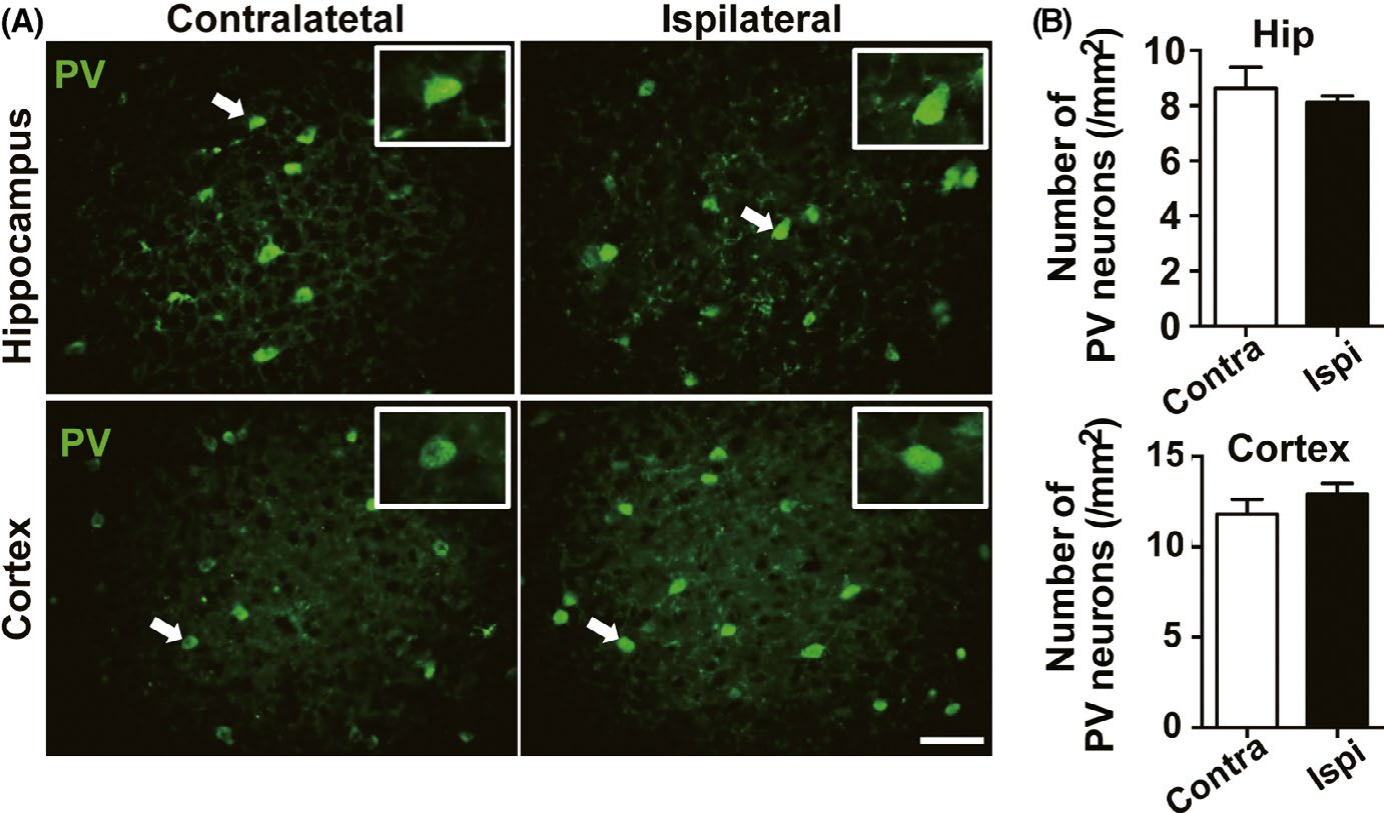
Pharmaco‐genetic activating parvalbumin neurons during epileptogenesis does not affect the number of parvalbumin neurons. A, Representative images of the parvalbumin^+^ neurons in the hippocampus and cortex from a *PV‐hM3Dq* mouse; scale bar 50 μm. Insert, enlarged views of parvalbumin^+^ neurons with white arrows. B, Statistics of the number of hippocampal and cortical parvalbumin^+^ neurons from 3 *PV‐hM3Dq* mice; Data are presented as means ± SEM

Next, we aimed to test the possible reason underlying the difference between pharmaco‐genetic control of parvalbumin neurons and pyramidal neurons. As consecutive activation of GABAergic neuron may lead to neuronal damage and thus produce limited anti‐seizure effect, to exclude this possibility, we performed immunohistochemical study and found that pharmaco‐genetic activating parvalbumin neurons during epileptogenesis did not affect the number of hippocampal and cortical parvalbumin neurons (Figure 4).

**FIGURE 5:**
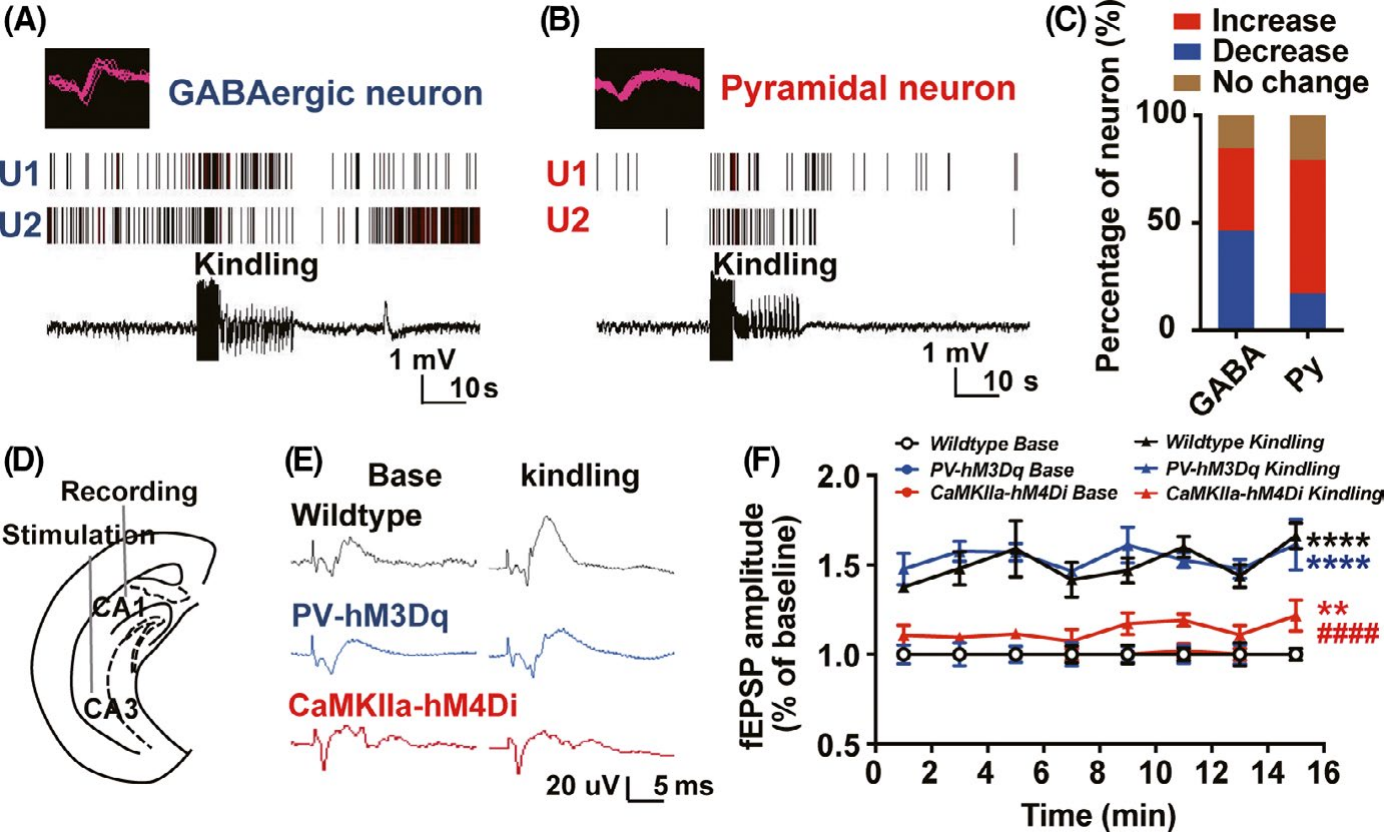
Pharmaco‐genetic inhibiting pyramidal neurons efficiently depotentiates hippocampal synaptic plasticity during epileptogenesis. A, B, Representative peri‐event raster histograms for the firing of putative GABAergic neuron (A) and pyramidal neurons (B) during hippocampal kindling‐induced seizures. C, Statistics of the responsive number of the putative GABAergic neuron and pyramidal neurons during hippocampal seizures from 4 wildtype mice. D, Experiment scheme for the fEPSP recording to measure hippocampal synaptic plasticity. E, Representative EEG recordings of the fEPSP during hippocampal kindling‐induced epileptogenesis from wildtype, *PV‐hM3Dq*, and *CaMKIIa‐hM4Di* mice, respectively. F, The effects of pharmaco‐genetic modulating parvalbumin or pyramidal neurons on the fEPSP during hippocampal kindling‐induced epileptogenesis in wildtype, *PV‐hM3Dq*, and *CaMKIIa‐hM4Di* mice, respectively. n = 5 for each group, **P < .01, ****P < .0001 compared with each base group, ^####^ *P* < .0001 compared with Wildtype Kindling group, two‐way ANOVA followed by post hoc Tukey test. Data are presented as means ± SEM

Then, we want to know how did the parvalbumin and pyramidal neurons respond to hippocampal kindling‐induced seizures in anesthetized mice. Single‐unit in vivo recordings showed that the firing rate of putative GABAergic neurons is greatly heterogeneous in response to kindling stimulation, 5/13 neurons increased their firing rate, 6/13 decreased, 2/13 had no change. While, firing rate of ~62% putative pyramidal neurons (18/29) increased their firing rate, remaining 5/29 decreased, 6/29 had no change (Figure 5A‐C). Consecutive activation of glutamatergic transmission easily induces synaptic plasticity, which is also one of the mechanisms underlying kindling‐induced epileptogenesis.^32^, ^33^ We further tested whether pharmaco‐genetic modulation of above two types of neurons would affect synaptic plasticity during epileptogenesis. Electrodes were stereotactically implanted into the ventral hippocampus for kindling stimulation and test stimulation, and into the CA1 for fEPSP recording (Figure 5D). We found that fEPSP amplitude was increased significantly in wildtype mice after kindling acquisition, suggesting enhanced synaptic plasticity during epileptogenesis (two‐way ANOVA followed by post hoc Tukey test, *F* = 164.5, *P* < .0001; Figure 5E,F). Interestingly, pharmaco‐genetic activating parvalbumin neurons did not affect fEPSP amplitude after kindling acquisition in *PV‐hM3Dq* mice (*P* = .8058 for PV‐hM3Dq kindling group vs Wildtype Kindling group), while pharmaco‐genetic inhibition of pyramidal neurons greatly reversed the kindling‐induced increased fEPSP amplitude in *CaMKIIa‐hM4Di* mice (*P* < .0001 for CaMKIIa‐hM4Di kindling group vs Wildtype Kindling group; Figure 5E,F). These above results demonstrated that pharmaco‐genetic inhibiting pyramidal neurons can reverse the enhanced synaptic plasticity during epileptogenesis.

## 4 DISCUSSION

Pharmaco‐genetics is emerging as a new alternative approach to control seizure in epilepsy. However, current pharmaco‐genetics for epilepsy treatment all focus on seizure control. In this study, we found that pharmaco‐genetic activating parvalbumin neurons with hM3Dq, which has been reported to increase the intracellular Ca^2+^ level by the CNO,^10^ limitedly retarded the progression of behavioral seizure stage and electrophysiological ADD during epileptogenesis induced by kindling. On the contrary, pharmaco‐genetic inhibiting pyramidal neurons with hM4Di, which has been reported to induce hyperpolarization by the CNO,^10^ robustly retarded the progression of seizure stages and ADD. Interestingly, activation of parvalbumin neurons delayed seizure development only in the early stage, but even accelerated seizure development in late stages. While, pharmaco‐genetic inhibiting pyramidal neurons produced an anti‐epileptogenic effect in both early and late stages. Here, we demonstrated that pharmaco‐genetics targeting pyramidal neurons, rather than parvalbumin neurons, efficiently retards hippocampal kindling‐induced epileptogenesis. As epileptogenesis can be induced by a variety of acute brain insults,^34^ whether pharmaco‐genetic targeting pyramidal neurons is generally applied in other type of epileptogenesis is worthy to be studied. Currently, there is no effective treatment to prevent the development epilepsy in patients at risk (epileptogenesis). Although previous studies showed that low‐frequency stimulation seems to be a relative safe treatment to retard epileptogenesis,^35^, ^36^ it is still not circuit‐specific and cell‐type‐specific. However, pharmaco‐genetic technology makes it possible to modulate selected cell populations at specific times while opening avenues for understanding of circuit mechanisms underlying epilepsy. Compared with optogenetics,^37^ it does not need implantation of biocompatible optical devices and represents a more valuable approach for epilepsy treatment in clinical translation.

Epileptogenesis is now considered as a circuit‐level syndrome pathologically characterized by “excitation‐inhibition” imbalance, caused by neuronal loss, synaptic reorganization and circuit rewiring.^38^, ^39^, ^40^ Specially, long‐term potentiation in glutamatergic transmission plays an important role in epileptogenesis.^41^ We verified that fEPSP amplitude increased significantly in wildtype mice after kindling acquisition, suggesting enhanced synaptic plasticity during epileptogenesis. Interestingly, pharmaco‐genetic activating parvalbumin neurons did not affect fEPSP amplitude, while pharmaco‐genetic inhibiting pyramidal neurons greatly reversed the kindling‐induced increased fEPSP amplitude, indicating that pharmaco‐genetic inhibiting pyramidal neurons, but not pharmaco‐genetic activating parvalbumin neurons, is able to reverse the enhanced synaptic plasticity during epileptogenesis. This can be due to the increased firing of majority of pyramidal neurons in response to kindling‐induced seizures demonstrated in our single‐unit recording experiment, as consecutive activation of glutamatergic transmission easily induces synaptic plasticity,^32^ which is also one of the mechanisms underlying kindling‐induced epileptogenesis.^33^ However, firing rates of GABAergic neurons are greatly heterogeneous in response to kindling and optogenetic induced seizures.^42^ Thus, inhibition of increased firing of pyramidal neurons during seizure may be more efficient in retarding kindling‐induced epileptogenesis and potentiation of synaptic plasticity.

Interestingly, pharmaco‐genetic activating parvalbumin neurons efficiently alleviated the EEG intensity of hippocampal seizures in our study. Meanwhile, studies from our lab and others reported that pharmaco‐genetic activating parvalbumin neurons can attenuate the severity of seizure in hippocampal kindled status and other in vivo and in vitro seizure models.^18^, ^19^, ^23^ However, pharmaco‐genetic activating parvalbumin neurons had only limited effect on the progression of seizure stage and ADD during epileptogenesis induced by kindling. It even accelerated seizure development in late stages. This discrepancy may be due to the different microcircuit mechanisms of GABAergic neurons underlying epileptogenesis and epileptic seizures. GABAergic transmission usually shows double‐edged function in epilepsy.^43^ In normal conditions, GABAergic neurons play an inhibitory role on excitatory pyramidal neurons, however, once the epileptogenesis starts, it would gradually make parvalbumin neuron easily to became depolarized in irreversible “pathology‐dependent” manner which is related to the dysfunction of chloride transporters.^26^, ^44^ This character of GABAergic neurons was also inferred as the clue to the fact that all currently available antiepileptic drugs are symptomatically in epileptic seizure control but not preventing epileptogenesis.^45^ Although pharmaco‐genetic activating parvalbumin neurons did not affect the number of parvalbumin neurons during epileptogenesis, cumulative pharmaco‐genetic activating parvalbumin neuron may also make function of the parvalbumin neuron easily to become depolarized in “activity‐dependent” manner due to the long‐term activation of chloride transporters.^46^ Pharmaco‐genetic activating PV neurons might also cause the disinhibition of GABAergic neurons on pyramidal neurons. Meanwhile, the majority of neurons are glutamatergic neurons in the hippocampus, the inferior position in cell numbers combined with the character of neuronal function may make the pharmaco‐genetic activating parvalbumin neurons to have limited anti‐epileptogenic effect or produce seizure‐promoting effects. However, undoubtedly, there also exist some limitations in our study. For example, only the hippocampal kindling model was used in our study. To enhance the translational significance, future studies should be applied on other well‐established epilepsy models to confirm the therapeutic value of Pharmaco‐genetic modulation of focal parvalbumin/pyramidal neurons on epileptogenesis. Meanwhile, whether Pharmaco‐genetic modulating neurons of seizure focus exerts effect on other epilepsy comorbidities such as memory deficits et al also needs to be further investigated.

In conclusion, our results demonstrated that pharmaco‐genetic inhibiting pyramidal neurons efficiently retard hippocampal kindling‐induced epileptogenesis and reverses the enhanced synaptic plasticity during epileptogenesis, compared with that of pharmaco‐genetic activating parvalbumin neurons, suggesting that pharmaco‐genetics targeting pyramidal neurons may be a promising and alternative approach for treating epileptogenesis.

## CONFLICT OF INTEREST

The authors declare no conflict of interest.
